# Bridging the gap: aligning economic research with disease burden

**DOI:** 10.1136/bmjgh-2021-005673

**Published:** 2021-06-07

**Authors:** Lauren A Do, Patricia G Synnott, Siyu Ma, Daniel A Ollendorf

**Affiliations:** 1 Center for the Evaluation of Value and Risk in Health, Tufts Medical Center, Boston, Massachusetts, USA; 2 HEOR, GlaxoSmithKline, Philadelphia, Pennsylvania, USA; 3 Tufts University School of Medicine, Boston, Massachusetts, USA

**Keywords:** health policies and all other topics, health economics, health systems, indices of health and disease and standardisation of rates, infections, diseases, disorders, injuries

## Abstract

**Introduction:**

Cost-effectiveness analysis (CEA) is critical for identifying high-value interventions that address significant unmet need. This study examines whether CEA study volume is proportionate to the burden associated with 21 major disease categories.

**Methods:**

We searched the Tufts Medical Center CEA and Global Health CEA Registries for studies published between 2010 and 2019 that measured cost per quality-adjusted life-year or cost per disability-adjusted life-year (DALY). Stratified by geographical region and country income level, the relationship between literature volume and disease burden (as measured by 2019 Global Burden of Disease estimates of population DALYs) was analysed using ordinary least squares linear regression. Additionally, the number of CEAs per intervention deemed ‘essential’ for universal health coverage by the Disease Control Priorities Network was assessed to evaluate how many interventions are supported by cost-effectiveness evidence.

**Results:**

The results located below the regression line but with relatively high burden suggested disease areas that were ‘understudied’ compared with expected study volume. Understudied disease areas varied by region. Higher-income and upper-middle-income country (HUMIC) CEA volume for non-communicable diseases (eg, mental/behavioural disorders) was 100-fold higher than that in low-income and lower-middle-income countries (LLMICs). LLMIC study volume remained concentrated in HIV/AIDS as well as other communicable and neglected tropical diseases. Across 60 essential interventions, only 33 had any supporting CEA evidence, and only 21 had a decision context involving a low-income or middle-income country. With the exception of one intervention, available CEA evidence revealed the 21 interventions to be cost-effective, with base-case findings less than three times the GDP per capita.

**Conclusion:**

Our analysis highlights disease areas that require significant policy attention. Research gaps for highly prevalent, lethal or disabling diseases, as well as essential interventions may be stifling potential efficiency gains. Large research disparities between HUMICs and LLMICs suggest funding opportunities for improving allocative efficiency in LLMIC health systems.

**Key questions**

**What is already known?**

- Cost-effectiveness analyses can help to guide policy-makers, payers and providers on coverage decisions by offering insights into the relative cost per unit of health gained of different health internventions.
- The volume of cost-effectiveness analysis literature has grown considerably over the past several decades, and captures a wide variety of diseases, interventions, countries and populations.

**What are the new findings?**

- Our findings indicate substantial differences in terms of available cost-effectiveness analysis studies between high-income and upper-middle-income countries and low-income and lower-middle income countries where current literature is disproportionately focused on higher-income settings.
- Relative to the burden they impose, some disease areas were found to be particularly ‘understudied’ by cost-effectiveness analysis, and these understudied disease areas differed by region and country-income level.
- Despite the clinical value of essential inventions that could form a universal healthcare package, the level of cost-effectiveness evidence focused on those interventions in low-income and middle-income countries is lacking.

**What do the new findings imply?**

- Closing the cost-effectivness research gap between higher-income and lower-income countries can aid in improving global health outcomes, especially in lower-income countries that face major budget constraints.
- By measuring the shifting nature of disease burden and priortising high value care, decision-makers will be better equipped to make adoption decisions that maximise the health benefits and cost savings of their respective populations.

## Introduction

Shifts in Global Burden of Disease (GBD) and times of crisis—such as the COVID-19 pandemic—can reshape health system priorities and signal a need for context-sensitive, evidence-based policy-making. Although global health outcomes have steadily improved over the last 30 years, growing and ageing populations, as well as expanded emphasis on non-communicable diseases and injuries, have applied continual pressure on clinicians, caretakers and patients alike.^1^ In order to alleviate disabling health outcomes, policy-makers must anticipate and adapt to these changing health service demands. Cost-effectiveness analysis (CEA) is an effective prioritisation tool for meeting these demands by identifying high-value interventions that combat diseases contributing the greatest burden in a particular setting.

CEA is commonly used by high-income countries to make decisions about efficient resource allocation. In recent years, the number of published CEAs emerging from low-income and middle-income countries (LMICs) has also accelerated as global initiatives focused on improving allocative efficiency and health gain momentum.^2^ CEA outcome measures such as cost per quality-adjusted life-year (QALY) gained or cost per disability-adjusted life-year (DALY) averted help researchers measure the value for money of health interventions by quantifying how such interventions affect quality and length of life, as well as the opportunity costs associated with opting for a given intervention or programme.

Economic evidence, including CEAs, can be especially beneficial for LMICs where access to quality healthcare is highly dependent on its affordability.^3^ With fewer resources than high-income countries, it is imperative that policy-makers in LMICs allocate funds towards care with the highest value–clinically and economically. Practically speaking, CEAs can be used to inform priority-setting for achieving universal health coverage (UHC).^4^ Efforts to expand UHC are predicated on the notion that all residents can gain affordable access to essential health services. In fact, both as a solution and ideology, UHC is a prominent feature of the 2030 Sustainable Development Goals endorsed by the United Nations.^5^

The objective of this study was to (1) analyse the relationship between CEA literature volume and disease burden; (2) explore how research gaps vary by geographical region and country income level and (3) examine whether cost-effectiveness evidence supports a list of interventions deemed ‘essential’ for UHC.

## Methods

### Data sources and inclusion criteria

Published CEA literature was identified using two databases: the CEA Registry,^6^ and the Global Health CEA Registry.^7^ Both databases are maintained by the Center for the Evaluation of Value and Risk in Health at Tufts Medical Center in Boston, Massachusetts, USA. The CEA Registry houses information on published cost-per-QALY studies while the Global Health CEA Registry contains cost-per-DALY literature. For the purpose of this study, we did not consider this distinction between QALYs and DALYs when summing the number of CEA studies. At the time of analysis, the registries contained comprehensive information on English-language CEAs published from 1976 to 2019. Collectively, there is information on nearly 29000 ratios and 35000 utility or disability weights for nearly 10000 studies. Articles summarised in both registries undergo a formalised review protocol that includes an extensive systematic literature search to identify relevant CEAs. The Registry team searches PubMed, Scopus, Ovid and Embase using broad, predefined search terms in order to capture the most comprehensive list of published CEAs as possible.^6^ To ensure that we prioritised the most relevant evidence, we included CEA studies that were published from 2010 to 2019.

Data on disease burden were obtained through the GBD, injuries and risk factors study conducted by the Institute for Health Metrics and Evaluation.^8^ The study provides DALY estimates for a comprehensive list of 369 diseases and injuries across 204 countries and territories.^1^ Disease burden was measured in DALYs incurred by the population at the country level in 2019, which was the most recently published GBD dataset available. DALYs were stratified by the GBD study super region classification system^8^ as well as the World Bank country income levels.^9^ The super regions (ie, Europe and Central Asia; Latin America and the Caribbean; North Africa and the Middle East; South Asia; Southeast Asia, East Asia and Oceania; and sub-Saharan Africa) and country income levels (ie, low-income, lower-middle-income, higher-middle-income and high-income country) were based on 2019 categorisations. The ‘high-income’ super region was excluded since this region is similarly captured by the World Bank income level categorisation. We consolidated the World Bank country income levels into two main plots: higher-income and upper-middle-income countries (HUMICs) and low-income and lower-middle-income countries (LLMICs).

Within each super region and country-income level, we further stratified disease burden by GBD ‘level 2 causes’. The 369 diseases used in the GBD study are classified using a hierarchical nested system consisting of four different levels.^1 8^ The highest level (ie, level 1) is comprised of the three broadest causes of death and disability (ie, communicable, maternal, neonatal and nutritional diseases; non-communicable diseases; injuries) and further increases in specificity up to level 4 (eg, cholera; major depressive disorder; endometriosis).^1^ Level 2 causes categorised all 369 diseases into 21 major disease areas (eg, neglected tropical diseases and malaria; neoplasms; transport injuries), which allowed us to identify therapeutic areas with relatively more or less cost-effectiveness evidence. Section 1 of online supplemental material provides a comprehensive list of disease categorisations.

10.1136/bmjgh-2021-005673.supp1 Supplementary data

Critical health interventions were identified using the essential UHC (EUHC) interventions flagged by the third Edition of the Disease Control Priorities (DCP-3) programme.^10^ We included 60 total interventions, representing sample interventions from each of the five platforms of care (ie, population based; community; health centre; first-level hospital; referral and specialty hospital) and each of the 21 programmes as categorised in EUHC (eg, surgery, maternal and neonatal health, cancer, etc). Additionally, all 21 GBD level 2 disease areas are represented, where 20 EUHC interventions are linked to a single disease, 20 interventions are linked to 2–5 diseases, and 20 interventions are linked to more than five diseases. A comprehensive description of all EUHC interventions included in this study is available in section 2 of the online supplemental material.

### Analysing the relationship between CEA literature volume and disease burden

We analysed the relationship between literature volume and disease burden by regressing the number of published CEA studies (cost-per-QALY plus cost-per-DALY) mapped to a GBD level 2 disease area against the corresponding disease burden (population DALYs) using ordinary least squares linear regression. Stratification was conducted for each of the six super regions and two income levels. Super region and country income assignments were based on the geographical setting of the CEA study (ie, the country-specific outcome measures (US$/QALY or US$/DALY) of the study as well as the location of the target population that the intervention was intended for). The data used in each CEA may have been collected directly in the study country, or extrapolated from a different setting, a common approach in LMIC-based studies.

To compare literature volume across disease areas, we categorised our study findings into ‘adequately studied’ and ‘understudied’ disease areas. As there is no widely accepted, absolute threshold for what is considered understudied or adequately studied, we defined these classifications by dividing each graphical plot into quadrants based on the midpoint of each regression line. As a result, a disease area that is deemed adequately studied in one setting may be considered understudied in another. Results located below the regression line but with relatively high burden (ie, the ‘southeast quadrant’) indicated disease areas that were understudied compared with expected study volume in that particular setting. Results located above the regression line, regardless of burden, were deemed relatively adequately studied.

### Measuring the CEA literature volume of critical health interventions

Relevant literature within the CEA and GH CEA Registries that evaluated at least one of the 60 interventions deemed essential for UHC was identified by matching a study’s intervention and corresponding disease area to that of an EUHC intervention description. Since each unique EUHC could be composed of multiple services that cover a multitude of diseases, a study was deemed relevant and accounted for it if it was included in at least one part of the EUHC intervention description. Study volume was also stratified by two different country income levels: HUMICs and LLMICs.

We then summarised the number of published studies focused on an EUHC intervention and whether the intervention was cost-effective. The base-case cost-effectiveness results were categorised using four common, country-specific willingness-to-pay thresholds: (1) cost saving, (2) less than one times gross domestic product (GDP) per capita (1×GDP per capita), (3) between one and three times the GDP per capita (1–3×GDP per capita) or (4) not cost-effective (ie, ratio exceeded three times GDP per capita (>3 xGDP per capita)).^11^

### Patient and public involvement

Neither patients nor the public were directly involved in the design, conduct, or reporting of the analyses in this study.

## Results

### Study characteristics

Across both registries, there were 7197 available studies published from 2010 to 2019: 91.7% (N=6602) of the total number of included studies came from the CEA Registry.

### Literature volume versus disease burden stratified by geographical region

**Figure 1:**
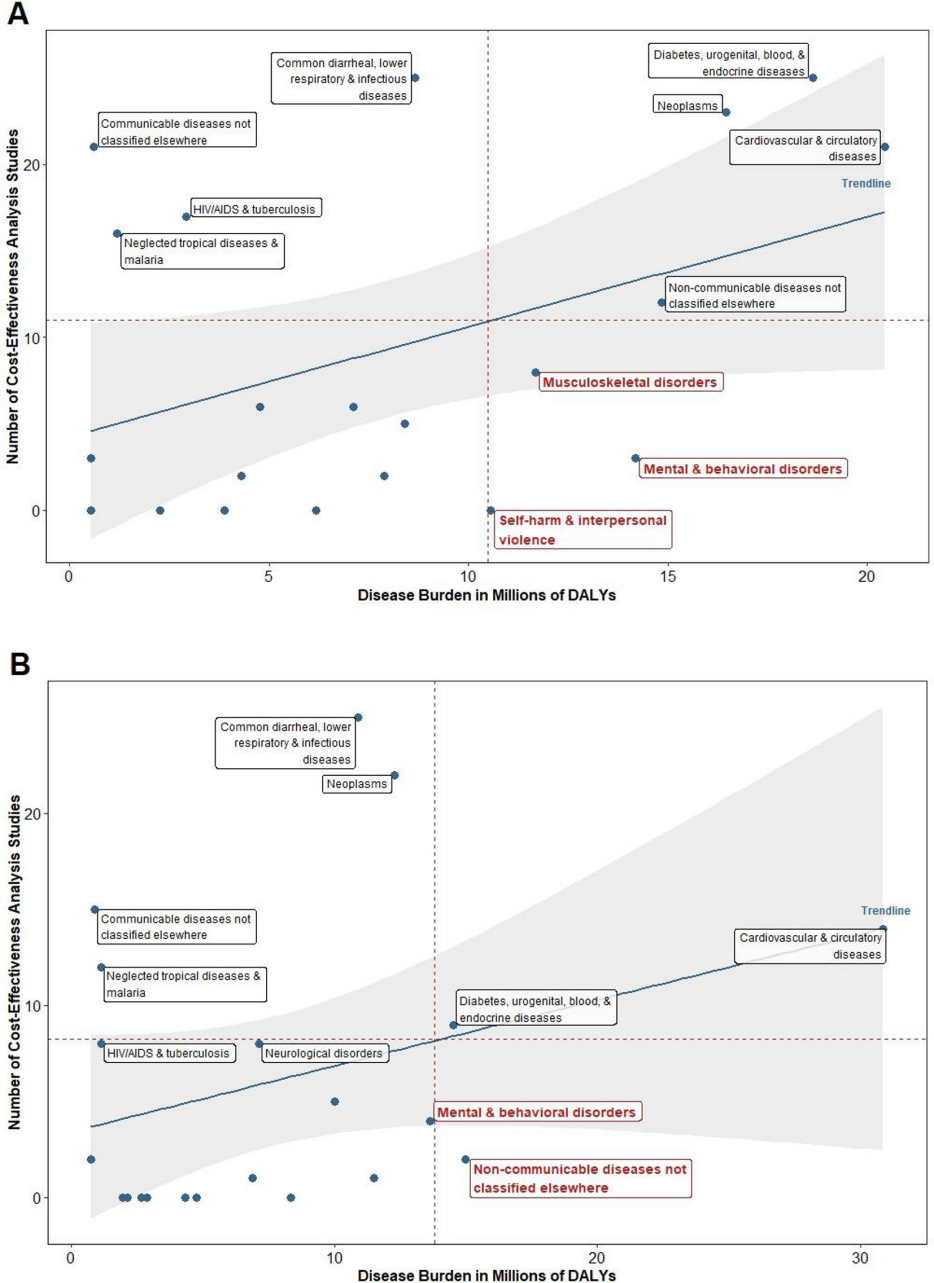
Number of CEAs versus disease burden for selected diseases: (A) Latin America and the Caribbean and (B) North Africa and the Middle East. CEA, cost-effectiveness analysis; DALYs, disability-adjusted life-years.

**Figure 2:**
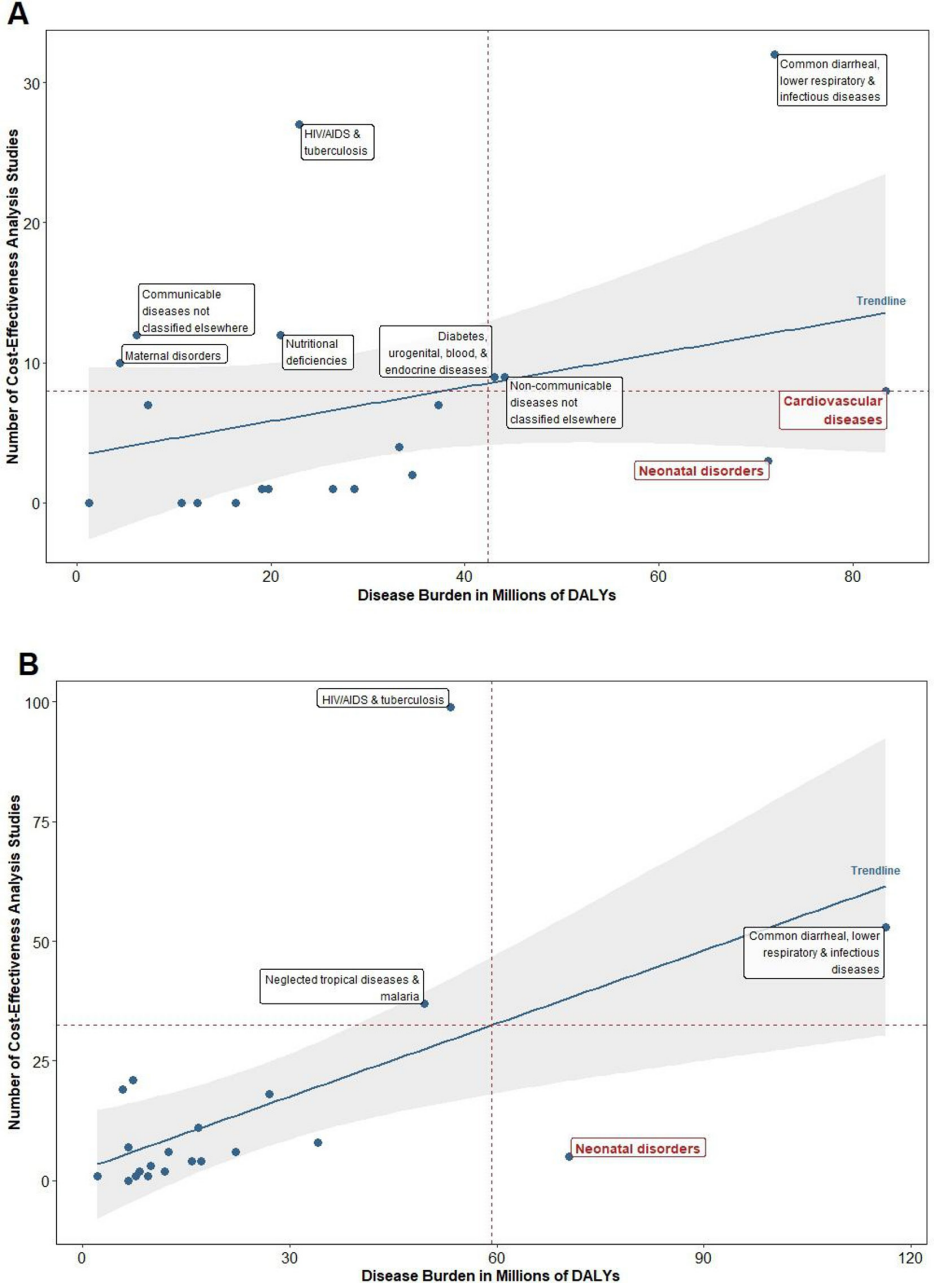
Number of CEAs versus disease burden for selected diseases: (A) South Asia and (B) sub-Saharan Africa. CEA, cost-effectiveness analysis; DALYs, disability-adjusted life-years.

Globally, mental and behavioural health (eg, depression, bipolar disorder, substance abuse) and neonatal disorders (eg, preterm birth complications, encephalopathy) were the most common understudied disease areas. Latin America and the Caribbean (see figure 1A), and North Africa and the Middle East (see figure 1B) were the two super regions with the lowest mental and behavioural health CEA coverage relative to population burden. For South Asia (see figure 2A) and sub-Saharan Africa (see figure 2B), neonatal disorders were the sole understudied disease area.

**Figure 3:**
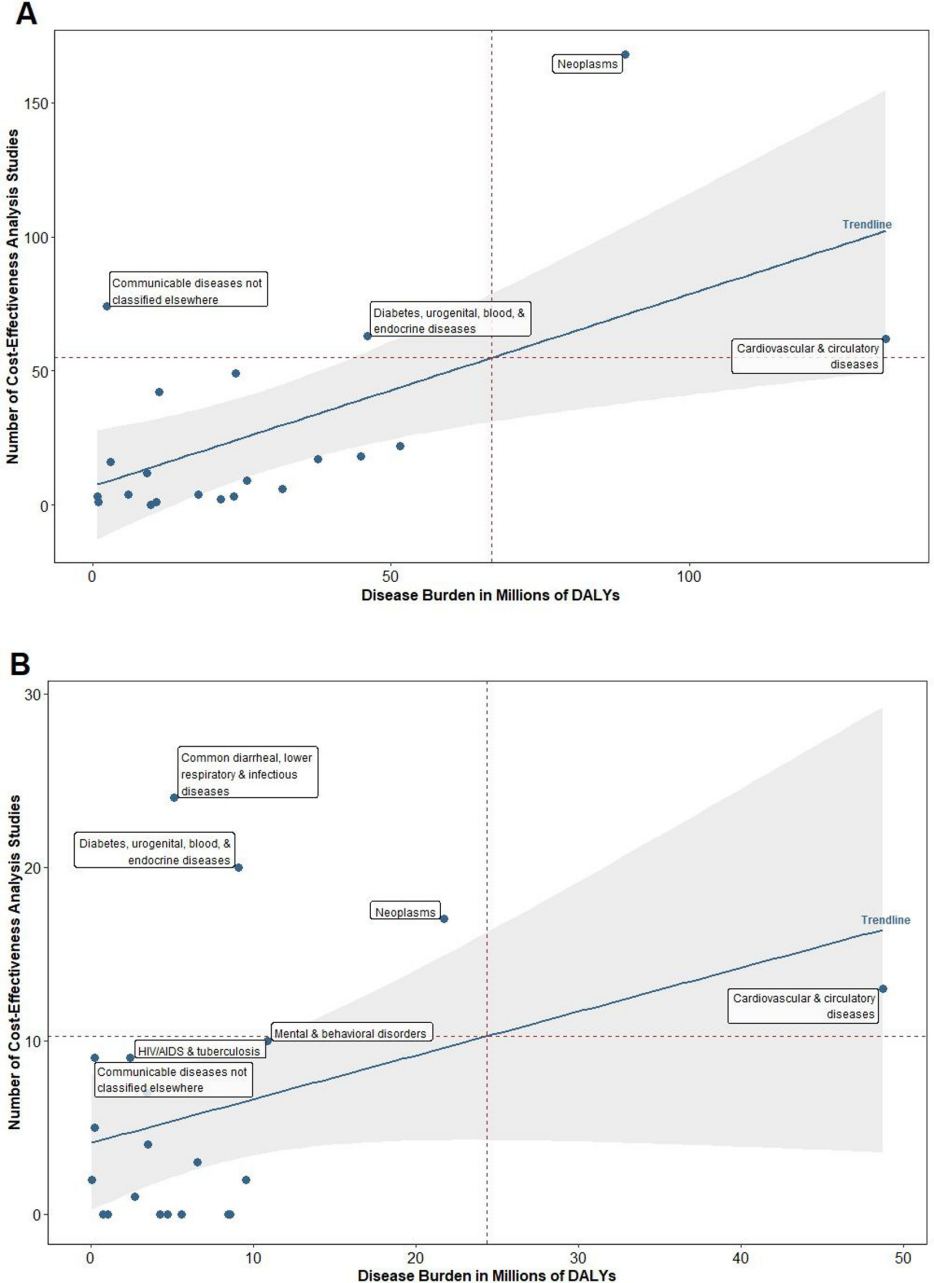
Number of CEAs versus disease burden for selected diseases: (A) Southeast Asia, East Asia and Oceania and (B) Europe and Central Asia. CEA, cost-effectiveness analysis; DALYs, quality-adjusted-life-years.

Southeast Asia, East Asia and Oceania (see figure 3A) as well as Europe and Central Asia (see figure 3B) had no understudied disease area. Cardiovascular and circulatory diseases (eg, ischaemic heart disease, intracerebral haemorrhage, ischaemic stroke) had study counts that were marginally higher than the quadrant line in each of these settings.

The most common adequately studied disease area was other ‘communicable diseases not classified elsewhere’ (eg, sexually transmitted diseases excluding HIV; acute hepatitis; leprosy; other infectious diseases not classified elsewhere), both on a global level and in four of the six super regions. Other common adequately studied-disease areas included HIV/AIDS and tuberculosis; diarrhoea, lower respiratory infections, meningitis, and other common infectious diseases; neglected tropical diseases and malaria. There were multiple disease areas that had as many studies as those within the ‘adequately studied’ category but imposed a much higher burden that excluded it from this category. For example, in North Africa and the Middle East, cardiovascular and circulatory diseases actually had more studies than its adequately-studied counterparts, but its population disease burden was nearly 30 times higher.

### Literature volume versus disease burden stratified by country income level

**Figure 4:**
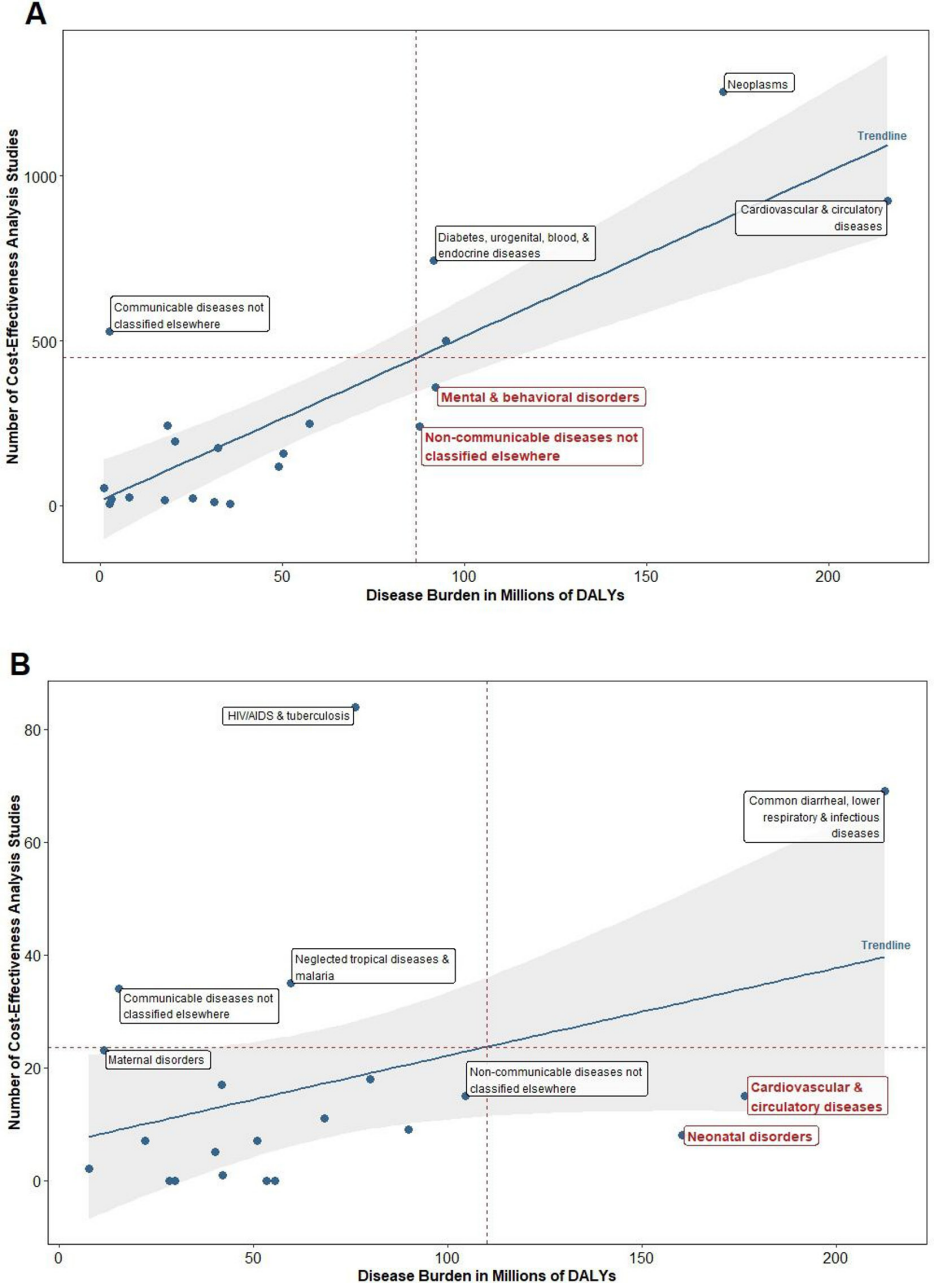
Number of CEAs versus disease burden for selected diseases: (A) High- and Upper-Middle-Income Countries and (B) Low- and Lower-Middle-Income Countries. CEA, cost-effectiveness analysis; DALYs, disability-adjusted life-years.

For HUMICs, ‘non-communicable diseases not classified elsewhere’ (eg, congenital anomalies; sense organ diseases; skin and subcutaneous diseases) and mental and behavioural health are the only two understudied disease areas (see figure 4A). Depression, anxiety and opioid addiction are the diseases with the highest level of burden within the mental and behavioural health category. For other non-communicable diseases, hearing loss, congenital heart anomalies and edentulous/severe tooth loss have the largest gaps in cost-effectiveness evidence. Other ‘communicable diseases not classified elsewhere’ (ie, syphilis; acute hepatitis b) is the only adequately-studied disease. Cardiovascular and circulatory diseases, and neoplasms have the highest study volume across all disease areas, but they contribute a significant amount of burden that is nearly 80 folds higher than other communicable diseases not classified elsewhere.

In LLMICs, cardiovascular and circulatory diseases as well as neonatal disorders are the most understudied disease areas (see figure 4B). These disease areas are dramatically understudied compared with HUMICs, given the very low volume of CEA publications in LLMICs. In fact, the highest number of studies for any given disease area is 84 for LLMICs vs 1256 for HUMICs. In LLMICs, heart disease, preterm birth, neonatal encephalopathy and intracerebral haemorrhages generate the greatest disease burden within those understudied disease areas. Adequately studied diseases for LLMICs were HIV/AIDs and tuberculosis; neglected tropical diseases and malaria; and other communicable diseases not classified elsewhere. HIV/AIDs and tuberculosis has over twice as many studies as neglected tropical diseases and malaria.

High-income and low-income countries do not share many similarities in terms of literature volume relative to disease burden. A clear example of this imbalance is in cardiovascular diseases. This disease area has one of the greatest disease burdens for both higher-income and lower-income settings, yet the number of studies focused on higher-income settings was 16 times the number of studies focused on lower-income countries while disease burden was only about 1.5 times greater.

### EUHC intervention CEA coverage and consistency

**Figure 5:**
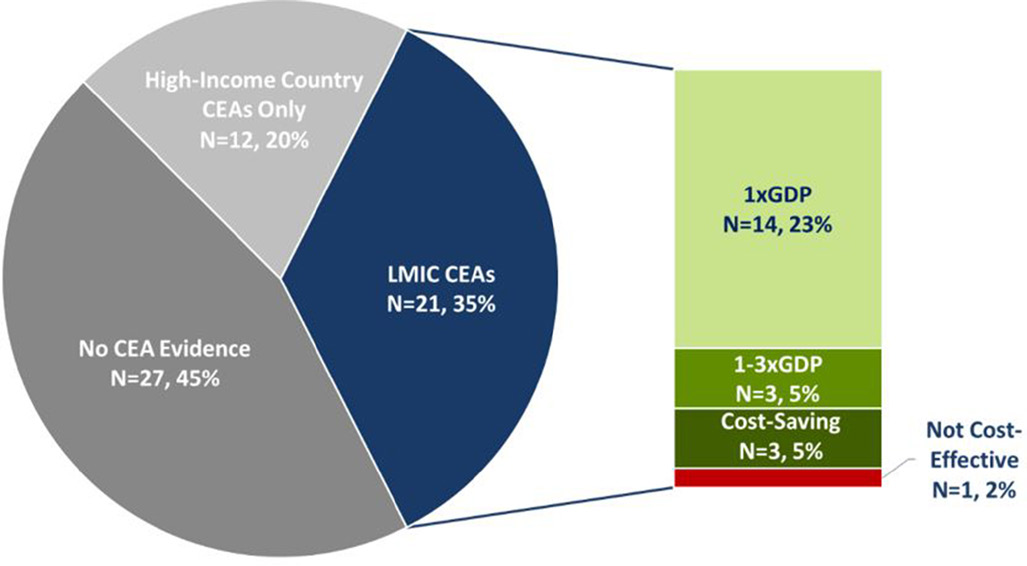
Pie chart represents the availability of at least one cost-effectiveness analysis (CEA) study for 60 essential universal health coverage (EUHC) interventions and organised by country of study. Associated stacked bar chart represents the distribution of base-case findings for studies that included a decision context involving an LMIC. LMIC, low-income and middle-income country.

Of the 60 EUHC interventions flagged by DCP-3, 55% (N=33) were associated with at least one published CEA study (see figure 5). Of these 33 interventions, there were 21 interventions (63.6%) in which the CEA’s decision context involved an LMIC; the remaining 12 interventions had CEAs that exclusively studied high-income settings. The number of available CEA studies for each intervention varied, ranging from 1 to 12 studies per intervention across both registries. The average was four studies for a single intervention.

Because the DCP-3 initiative was developed to meet the needs of LMICs, we focused our evidence review for the 21 interventions that were studied in such a setting. We found that with the exception of one intervention, available CEA evidence revealed those essential interventions to be cost-effective. Among these, 14.3% (N=3) were cost-saving, 66.7% (N=14) did not exceed a willingness-to-pay threshold of less than 1xGDP per capita, and 14.3% (N=3) did not exceed 1–3×GDP per capita. The one exception (ie,>3×GDP per capita) was a hospital-based surgical termination of pregnancy by manual vacuum aspiration (MVA), dilation or curettage, in comparison to clinic-based surgical abortions of the same methods.^12^

## Discussion

We examined the relationship between CEA study volume and disease burden and highlighted understudied diseases areas, and the essential interventions that address those diseases with the highest burden. Our findings indicate that the relationship between CEA study volume and disease burden differs across geographical regions, and that clear gaps remain in the level of CEA evidence available to address diseases with the greatest burden. These gaps are largely due to the persisting research disparities between higher-income and lower-income countries. Both share the same relative level of disease burden in terms of population DALYs, yet economic research in higher-income countries far surpasses that in lower-income countries. For example, cardiovascular diseases are the leading cause of death worldwide, with attributed global DALYs trending upward as a result of increased life expectancy.^13^ Yet, even though 80% of the global cardiovascular disease deaths occurred in LMICs between 1990 and 2019,^13 14^ CEAs studying cardiovascular disease interventions in higher-income countries outnumber those in lower-income settings by 16–1.

We also see this imbalance play out in the different metrics used in CEAs. Higher-income countries mainly use QALYs, whereas DALYs have been common in LMICs.^15^ Both metrics can measure quality and length of life. However, QALYs include considerations of health states and conditions rather than specific diseases, which makes it better suited to capture nuanced health preferences related to non-communicable or chronic diseases. DALYs, on the other hand, focus on impacts of disability and early death, which may better target the infectious diseases that are relatively more prevalent in LMICs.^16^ However, while LMICs experience a greater burden from communicable diseases compared with higher-income countries, this does not mitigate the significant and growing burden of non-communicable diseases in these settings. For example, adequately studied diseases for LMICs in our analysis included HIV/AIDS, tuberculosis and neglected tropical diseases, which suggests a heavy focus on communicable diseases even though only two of these countries (Ukraine and Lesotho) did not experience an epidemiological shift from communicable to non-communicable diseases between 1990 and 2019.^1^ And perhaps counterintuitively, countries with a lower Socio-Demographic Index—a measure of a country’s development with lower values representing less development—experienced greater shifts toward non-communicable diseases.^1^ Additionally, some may argue that DALYs are more suited for LMIC settings where available data on preference-based health utilities needed to conduct cost per QALY studies are limited or difficult to obtain.^17^ Collectively, these trends point to a need for LMICs to play ‘catch-up’ in terms of data and resources needed to further research the growing non-communicable disease burden.

Vast differences also appear across different geographical regions, Europe and Central Asia and Southeast Asia, East Asia and Oceania had no understudied diseases relative to burden, while Latin America and the Caribbean had as many as three. Only one-third of the plotted points across all regions represented a study volume of at least 10 CEAs. These research disparities were a major rationale for not making the quadrants fixed across all plots. It was also why we did not analyse a plot from a global perspective because the bulk of the literature is focused on high-income countries. So, the definition for ‘adequately studied’ and ‘understudied’ was dependent on the region, which set a much lower standard for LMICs in terms of research volume. This puts LMICs at a disadvantage. Although some diseases may be deemed ‘adequately studied’ in relative terms, they could in fact be grossly understudied when compared with settings with more resources.

Prior research examining literature volume against disease burden suggested that possible explanations for the presence of understudied diseases could be due to the limited funds and resources for conducting economic evaluations in LMICs.^2^ For example, in higher-income countries, the decision to economically evaluate interventions could be tied to financial incentives for pharmaceutical companies to demonstrate value, regardless of disease burden. These incentives may not be as prevalent in LMICs.^2^ In fact, the majority of the studies within the CEA Registry are focused on pharmaceutical interventions in high-income countries.^6^ Research priorities may also be related to the relative abundance of certain interventions.^18^ For example, organisations such as Gavi provides substantial funding to vaccination programmes in LMICs.^19^ This may contribute to higher proportions of CEAs related to communicable diseases and leaves non-communicable diseases relatively understudied.

Given limited resources, LMICs could use CEA to evaluate essential interventions that could form a UHC package.^20^ Even though a minority of the essential interventions evaluated in our analysis focused on an LMIC, those that did proved to be highly cost-effective. The one intervention deemed not-cost effective according to a single study was unrelated to the service itself (ie, surgical termination of pregnancy) but rather the clinical setting.^12^ DCP-3 suggested that surgical termination of pregnancies be performed in a first-level hospital setting.^10^ However, the 2009 CEA found hospital-based MVAs to be more expensive and clinically inferior to clinic-based MVAs. Dilation and curettage was also dominated by clinic-based MVAs. This particular EUHC service may still be cost-effective, but will be dependent on the setting in which it is offered. With few economic evaluations, there are limited opportunities for LMIC health systems to rank UHC interventions according to their value for money. This further leaves effective interventions without a good business case for why policy-makers in LMICs should invest in them.

For LMICs that lack research funding and capacity, stakeholders may find it tempting to extrapolate available evidence from economic studies conducted elsewhere to fit the needs of their particular setting. While this approach may seem faster and less costly than repeating the same study in a different setting, existing evidence in other settings may not be directly transferable or generalisable to LMICs due to differences in disease prevalence, local health preferences, clinical practices and availability of healthcare resources.^21^ Stakeholders must be cautious about examining the clinical and economic data employed by CEAs conducted elsewhere. Additionally, economic evaluations generally address the local preferences and standards of the study setting, which can be difficult to quantifiably measure and extrapolate.

We note some key limitations of our study. First, the CEA and Global Health CEA Registries contain only English-language studies, and are limited to published, peer-reviewed papers. With regard to language, recent analyses indicate that, within the last decade, over 95% of scientific citations were available in English, suggesting a minimal impact of excluding studies published solely in another language.^22^ Technical reports, health technology assessments and other grey literature were not included in our analysis; the full impact of such exclusions on our results is unknown. However, it is likely the case that the majority of such studies focus on an HUMIC decision context, which would have little effect on our estimates of LLMIC study volume. Moreover, the incremental gain from grey literature and non-English searches may not be sufficient to warrant the additional time and resources required to conduct a thorough search.^23^ Second, the EUHC intervention classifications we used do not adhere to a common structure, making it difficult to categorise using a standardised system such as the International Classification of Diseases (ICD-10) or GBD cause levels. For example, a single EUHC intervention may include multiple interventions for multiple target populations or diseases. As a result, we considered a CEA study ‘relevant’ if it’s intervention and disease characteristic matched some portion of an EUHC intervention description. As of yet, no initiative has effectively established an algorithm-based approach for linking UHC interventions to a system of standardised disease codes. Finally, the research gaps discussed in this study may be influenced by publication bias, in which stakeholders may be less inclined to fund studies that address very expensive interventions or publish unfavourable results. As an extensive amount of data are needed to properly measure publication bias,^24 25^ we were unable to explore the possibility of such bias across all countries, health interventions and diseases included in this study.

Given these limitations, future research could use more detailed disease categories to pinpoint specific diseases with the largest research gaps. Other possibilities include evaluating the quality of the studies that were identified and used within the analysis, and integrating non-English language and unpublished technical reports in a supplemental search.

## Conclusion

Improving health outcomes requires more than just measuring the prevalence of disease, but rather measuring the comparative burden of diseases, how burden shifts over time and prioritising high value care to address those diseases that are taxing health systems the most. CEA allows decision-makers to compare burden and costs across different types of diseases and interventions in order to align health system priorities with their respective populations. Our study has shown that there are economic research gaps that exist for the world’s most burdensome diseases. These gaps also reveal clear disparities between higher-incomed and lower-incomed settings in terms of available economic evidence. However, these gaps provide substantial opportunities for governments and philanthropic foundations to fund new research agendas that empower academic institutions to increase their local capacity to conduct CEAs. In the long run, policy-makers will be better equipped to make evidence-informed and context-sensitive decisions for their settings.

## Data Availability

The CEA Registry and the Global Health CEA Registry are held by the Center for the Evaluation of Value and Risk in Health at Tufts Medical Center, and are available at www.cearegistry.org and www.ghcearegistry.org, respectively. Data from the 2019 Global Burden of Diseases study is held by the Institute for Health Metrics and Evaluation and publicly available through www.healthdata.org/gbd/2019.
