# Supplementary material for: Bridging the gap: aligning economic research with disease burden
Source: BMJ Glob Health. 2021 Jun 7;6(6):e005673. doi: 10.1136/bmjgh-2021-005673 (PMC8186754; doi:10.1136/bmjgh-2021-005673)
Supplement: Supplementary data [file bmjgh-2021-005673supp001.pdf]

SUPPLEMENTARY MATERIALS

Section 1: Global Burden of Disease (GBD) Classification Levels

Level 1	Level 2	Level 3	Level 4
Communicable maternal, neonatal, and nutritional disorders	HIV/AIDS and tuberculosis	Tuberculosis	
		HIV/AIDS	HIV disease resulting in mycobacterial infection
			HIV disease resulting in other specified or unspecified diseases
	Diarrhea, lower respiratory infections, meningitis, and other common infectious diseases	Diarrheal diseases	Cholera
			Other salmonella infections
			Shigellosis
			Enteropathogenic E coli infection
			Enterotoxigenic E coli infection
			Campylobacter enteritis
			Amoebiasis
			Cryptosporidiosis
			Rotaviral enteritis
			Other diarrheal diseases
		Typhoid and paratyphoid fevers	
		Lower respiratory infections	Influenza
			Pneumococcal pneumonia
			Hemophilus influenza type B pneumonia
			Respiratory syncytial virus pneumonia
			Other lower respiratory infections
		Upper respiratory infections	
		Otitis media	
		Meningitis	Pneumococcal meningitis
			Hemophilus influenza type B meningitis
			Meningococcal infection
			Other meningitis

Do LA, *et al. BMJ Global Health* 2021; 6:e005673. doi: 10.1136/bmjgh-2021-005673

		Encephalitis	
		Diphtheria	
		Whooping cough	
		Tetanus	
		Measles	
		Varicella	
	Neglected tropical diseases and malaria	Malaria	
		Chagas disease	
		Leishmaniosis	
		African trypanosomiasis	
		Schistosomiasis	
		Cysticercosis	
		Echinococcosis	
		Lymphatic filariasis	
		Onchocerciasis	
		Trachoma	
		Dengue	
		Yellow fever	
		Rabies	
		Intestinal nematode infections	Ascariasis
			Trichuriasis
			Hookworm disease
		Food-borne trematodiases	
		Other neglected tropical diseases	
	Maternal disorders	Maternal hemorrhage	
		Maternal sepsis	
		Hypertensive disorders of pregnancy	
		Obstructed labor	
		Abortion	
		Other maternal disorders	
		Preterm birth complications	

	Neonatal disorders	Neonatal encephalopathy (birth asphyxia/trauma)	
		Sepsis and other infectious disorders of the newborn baby	
		Other neonatal disorders	
	Nutritional deficiencies	Protein-energy malnutrition	
		Iodine deficiency	
		Vitamin A deficiency	
		Iron-deficiency anemia	
		Other nutritional deficiencies	
	Other communicable, maternal, neonatal, and nutritional disorders	Sexually transmitted diseases excluding HIV	Syphilis
			Sexually transmitted chlamydial diseases
			Gonococcal infection
			Trichomoniasis
			Other sexually transmitted diseases
		Hepatitis	Acute hepatitis A
			Acute hepatitis B
			Acute hepatitis C
			Acute hepatitis E
		Leprosy	
		Other infectious diseases	
Non-communicable diseases	Neoplasms	Esophageal cancer	
		Stomach cancer	
		Liver cancer	Liver cancer secondary to hepatitis B
			Liver cancer secondary to hepatitis C
			Liver cancer secondary to alcohol use
			Other liver cancer
		Larynx cancer	
		Trachea, bronchus, and lung cancers	
		Breast cancer	
		Cervical cancer	

Do LA, *et al. BMJ Global Health* 2021; 6:e005673. doi: 10.1136/bmjgh-2021-005673

		Uterine cancer	
		Prostate cancer	
		Colon and rectum cancers	
		Mouth cancer	
		Nasopharynx cancer	
		Cancer of other part of pharynx and oropharynx	
		Gallbladder and biliary tract cancer	
		Pancreatic cancer	
		Malignant melanoma of skin	
		Non-melanoma skin cancer	
		Ovarian cancer	
		Testicular cancer	
		Kidney and other urinary organ cancers	
		Bladder cancer	
		Brain and nervous system cancers	
		Thyroid cancer	
		Hodgkin's disease	
		Non-Hodgkin lymphoma	
		Multiple myeloma	
		Leukemia	
		Other neoplasms	
	Cardiovascular and circulatory diseases	Rheumatic heart disease	
		Ischemic heart disease	
		Cerebrovascular disease	Ischemic stroke
			Hemorrhagic and other non-ischemic stroke
		Hypertensive heart disease	
		Cardiomyopathy and myocarditis	
		Atrial fibrillation and flutter	
		Aortic aneurysm	
		Peripheral vascular disease	

Do LA, *et al. BMJ Global Health* 2021; 6:e005673. doi: 10.1136/bmjgh-2021-005673

		Endocarditis	
		Other cardiovascular and circulatory diseases	
	Chronic respiratory diseases	Chronic obstructive pulmonary disease	
		Pneumoconiosis	
		Asthma	
		Interstitial lung disease and pulmonary sarcoidosis	
		Other chronic respiratory diseases	
	Cirrhosis of the liver	Cirrhosis of the liver secondary to hepatitis B	
		Cirrhosis of the liver secondary to hepatitis C	
		Cirrhosis of the liver secondary to alcohol use	
		Other cirrhosis of the liver	
	Digestive diseases (except cirrhosis)	Peptic ulcer disease	
		Gastritis and duodenitis	
		Appendicitis	
		Paralytic ileus and intestinal obstruction without hernia	
		Inguinal or femoral hernia	
		Non-infective inflammatory bowel disease	
		Vascular disorders of intestine	
		Gallbladder and bile duct disease	
		Pancreatitis	
		Other digestive diseases	
	Neurological disorders	Alzheimer’s disease and other dementias	
		Parkinson’s disease	
		Epilepsy	
		Multiple sclerosis	

		Migraine	
		Tension-type headache	
		Other neurological disorders	
	Mental and behavioral disorders	Schizophrenia	
		Alcohol use disorders	
		Drug use disorders	Opioid use disorders
			Cocaine use disorders
			Amphetamine use disorders
			Cannabis use disorders
			Other drug use disorders
		Unipolar depressive disorders	Major depressive disorder
			Dysthymia
		Bipolar affective disorder	
		Anxiety disorders	
		Eating disorders	
		Pervasive development disorders	Autism
			Asperger's syndrome
		Childhood behavioral disorders	Attention-deficit hyperactivity disorder
			Conduct disorder
		Idiopathic intellectual disability	
		Other mental and behavioral disorders	
	Diabetes, urogenital, blood, and endocrine diseases	Diabetes mellitus	
		Acute glomerulonephritis	
		Chronic kidney diseases	Chronic kidney disease due to diabetes mellitus
			Chronic kidney disease due to hypertension
			Chronic kidney disease unspecified
		Urinary diseases and male infertility	Tubulointerstitial nephritis, pyelonephritis, and urinary tract infections
			Urolithiasis
			Benign prostatic hyperplasia
			Male infertility

			Other urinary diseases
		Gynecological diseases	Uterine fibroids
			Polycystic ovarian syndrome
			Female infertility
			Endometriosis
			Genital prolapse
			Premenstrual syndrome
			Other gynecological diseases
		Hemoglobinopathies and hemolytic anemias	Thalassemia
			Sickle-cell disorders
			G6PD deficiency
			Other hemoglobinopathies and hemolytic anemias
		Other endocrine, nutritional, blood, and immune disorders	
	Musculoskeletal disorders	Rheumatoid arthritis	
		Osteoarthritis	
		Low back and neck pain	
		Low back pain	
		Neck pain	
		Gout	
		Other musculoskeletal disorders	
	Other non-communicable diseases	Congenital anomalies	Neural tube defects
			Congenital heart anomalies
			Cleft lip and cleft palate
			Down’s syndrome
			Other chromosomal abnormalities
			Other congenital anomalies
		Skin and subcutaneous diseases	Eczema
			Psoriasis
			Cellulitis

Do LA, *et al. BMJ Global Health* 2021; 6:e005673. doi: 10.1136/bmjgh-2021-005673

			Abscess, impetigo, and other bacterial skin diseases
			Scabies
			Fungal skin diseases
			Viral skin diseases
			Acne vulgaris
			Alopecia areata
			Pruritus
			Urticaria
			Decubitus ulcer
			Other skin and subcutaneous diseases
		Sense organ diseases	Glaucoma
			Cataracts
			Macular degeneration
			Refraction and accommodation disorders
			Other hearing loss
			Other vision loss
			Other sense organ diseases
			Oral disorders
			Dental caries
			Periodontal disease
			Edentulism
		Sudden infant death syndrome	
Injuries	Transport injuries	Road injury	Pedestrian injury by road vehicle
			Pedal cycle vehicle
			Motorized vehicle with two wheels
			Motorized vehicle with three or more wheels
			Road injury other
		Other transport injury	
		Falls	
		Drowning	

Do LA, *et al. BMJ Global Health* 2021; 6:e005673. doi: 10.1136/bmjgh-2021-005673

	Unintentional injuries other than transport injuries	Fire, heat, and hot substances	
		Poisonings	
		Exposure to mechanical forces	Mechanical forces (firearm)
			Mechanical forces (other)
		Adverse effects of medical treatment	
		Animal contact	Animal contact (venomous)
			Animal contact (non-venomous)
		Unintentional injuries not classified elsewhere	
	Self-harm and interpersonal violence	Self-harm	
		Interpersonal violence	Assault by firearm
			Assault by sharp object
			Assault by other means
	Forces of nature, war, and legal intervention	Exposure to forces of nature	
		Collective violence and legal intervention	

Section 2: Selected Essential Universal Healthcare Interventions by DCP3 included in the study

DCP3 code	DCP3 intervention name	Associated GBD causes	Platform	Identified LMIC CEA Evidence	Identified high-income CEA Evidence
C34	Conduct larviciding and water-management programs in high malaria transmission areas where mosquito breeding sites can be identified and regularly targeted	Malaria; Dengue; Zika; Encephalitis; Yellow fever	Community	*	
C35	In all malaria-endemic countries, diagnosis with rapid test or microscopy (including speciation) followed by	Malaria	Community	*	

	treatment with ACTs (or current first-line combination)				
C4	Promotion of breastfeeding or complementary feeding by lay health workers	Nutritional deficiencies; Neonatal sepsis and other neonatal infections	Community		
C43	Early detection and treatment of Chagas disease, human African trypanosomiasis, leprosy, and leishmaniosis	Chagas disease; African trypanosomiasis; Leprosy; Leishmaniasis	Community	*	
C47	Exercise-based pulmonary rehabilitation for patients with obstructive lung disease	Chronic respiratory diseases	Community		*
C50	Parent training for high-risk families, including nurse home visitation for child maltreatment	Interpersonal violence	Community	*	
C51	WASH behavior change interventions, such as community-led total sanitation	Enteric infections	Community		*
C52	Cardiac and pulmonary rehabilitation programs	Chronic respiratory diseases; Rheumatic heart disease; Ischemic heart disease; Hypertensive heart disease; Non-rheumatic valvular heart disease; Cardiomyopathy and myocarditis; Atrial fibrillation and flutter; Aortic aneurysm; Peripheral artery disease; Endocarditis; Other cardiovascular and circulatory diseases	Community		*
C53	Early childhood development rehabilitation interventions, including	Autism spectrum disorders; Attention-deficit/hyperactivity disorder; Conduct disorder;	Community		

Do LA, *et al. BMJ Global Health* 2021; 6:e005673. doi: 10.1136/bmjgh-2021-005673

	motor, sensory, and language stimulation	Idiopathic developmental intellectual disability			
C56	Pressure area prevention and supportive seating interventions for wheelchair users	Injuries, Stroke; Musculoskeletal disorders; Parkinson's disease; Multiple sclerosis; Motor neuron disease	Community		
C58	Training and retraining for disorders of speech, swallowing, communication, and cognition	Stroke; Parkinson's disease; Multiple sclerosis; Motor neuron disease	Community		*
C59	Training, retraining, and exercise programs that address musculoskeletal injuries and disorders, including chronic low back and neck pain	Injuries; Musculoskeletal disorders	Community		*
FLH1	Detection and management of fetal growth restriction	Protein-energy malnutrition; Other maternal disorders; Other neonatal disorders	First-level hospital		
FLH10	Surgical termination of pregnancy by annual vacuum aspiration, dilation and curettage	Maternal abortion and miscarriage	First-level hospital	*	
FLH11	Full supportive care for severe childhood infections with danger signs	Lower respiratory infections; Urinary tract infections; Diarrheal diseases; Malaria; HIV/AIDS; Drug-susceptible tuberculosis; Multidrug-resistant tuberculosis without extensive drug resistance; Extensively drug-resistant tuberculosis; Zika; Dengue; Ebola	First-level hospital		
FLH12	Management of severe acute malnutrition associated with serious infection	Protein-energy malnutrition	First-level hospital	*	

FLH13	Early detection and treatment of early-stage cervical cancer	Cervical cancer	First-level hospital		*
FLH2	Induction of labor post-term	Maternal hemorrhage; Other maternal disorders; Maternal sepsis and other maternal infections; Maternal hypertensive disorders; Maternal obstructed labor and uterine rupture; Maternal abortion and miscarriage; Ectopic pregnancy; Indirect maternal deaths; Late maternal deaths; Neonatal encephalopathy due to birth asphyxia and trauma	First-level hospital		
FLH20	Management of acute coronary syndromes with aspirin, unfractionated heparin, and generic thrombolytic (when indicated)	Ischemic heart disease	First-level hospital	*	
FLH22	Management of acute exacerbations of asthma and COPD using systemic steroids, inhaled beta-agonists, and, if indicated, oral antibiotics and oxygen therapy	Chronic obstructive pulmonary disease; Asthma	First-level hospital		*
FLH27	In settings where sickle cell disease is a public health concern, universal newborn screening followed by standard prophylaxis against bacterial infections and malaria	Sickle cell disorders; Neonatal sepsis and other neonatal infections; Malaria	First-level hospital	*	
FLH46	Basic skin grafting	Fire, heat, and hot substances; Malignant skin melanoma; Decubitus ulcer	First-level hospital		

FLH15	Management of maternal sepsis, including early detection at health centers	Maternal sepsis and other maternal infections	First-level hospital	*	
FLH50	Tube thoracostomy	Tracheal, bronchus, and lung cancer; Transport injuries; Unintentional injuries; Exposure to forces of nature; Other unintentional injuries; Exposure to mechanical forces; Foreign body; Self-harm and interpersonal violence.	First-level hospital		
FLH52	Compression therapy for amputations, burns, and vascular or lymphatic disorders	Fire, heat, and hot substances; Lymphatic filariasis	First-level hospital		
FLH53	Evaluation and acute management of swallowing dysfunction	Stroke; Lip and oral cavity cancer; Nasopharynx cancer; Other pharynx cancer; Esophageal cancer	First-level hospital		
FLH54	Fabrication, fitting, and training in the use of prosthetics, orthotics, and splints	Transport injuries; Unintentional injuries; Exposure to forces of nature; Other unintentional injuries; Exposure to mechanical forces; Foreign body; Self-harm and interpersonal violence	First-level hospital		*
H17	Referral of cases of treatment failure for drug susceptibility testing; enrollment of those with MDR-TB for treatment per WHO guidelines (either short or long regimen)	Multidrug- resistant tuberculosis without extensive drug resistance; HIV/AIDS; Multidrug-resistant tuberculosis without extensive drug resistance	First-level hospital		

H36	Fracture reduction and placement of external fixator and use of traction for fractures	Transport injuries; Unintentional injuries; Exposure to forces of nature; Other unintentional injuries; Exposure to mechanical forces; Foreign body; Self-harm and interpersonal violence	First-level hospital		*
HC1	Early detection and treatment of neonatal pneumonia with oral antibiotics	Lower respiratory infections; Neonatal sepsis and other neonatal infections	Health center		
HC13	Among all individuals who are known to be HIV positive, immediate ART initiation with regular monitoring of viral load for adherence and development of resistance	HIV/AIDS	Health center	*	
HC14	Psychological treatment for mood, anxiety, ADHD, and disruptive behavior disorders	Depressive disorders; Bipolar disorder; Anxiety disorders; Attention-deficit/hyperactivity disorder; Conduct disorder	Health center	*	
HC15	Management of complications following FGM	Sexual violence	Health center		
HC16	Post gender-based violence care, including counselling, provision of emergency contraception, and rape-response referral (medical and judicial)	HIV/AIDS and sexually transmitted infections; Depressive disorders; Anxiety disorders	Health center		
HC19	For individuals testing positive for hepatitis B and C, assessment of treatment eligibility by trained providers followed by initiation and monitoring of antiviral treatment when indicated	Acute hepatitis B; Acute hepatitis C; Cirrhosis and other chronic liver diseases due to hepatitis B; Cirrhosis and other chronic liver diseases due to hepatitis C; Liver cancer due to hepatitis B; Liver cancer due to hepatitis C	Health center	*	

HC23	Provider-initiated testing and counselling for HIV, STIs, and hepatitis, for all in contact with health system in high- prevalence settings, including prenatal care with appropriate referral or linkage to care including immediate ART initiation for those testing positive for HIV	HIV/AIDS and sexually transmitted infections; acute hepatitis B; Acute hepatitis C; Cirrhosis and other chronic liver diseases due to hepatitis B; Cirrhosis and other chronic liver diseases due to hepatitis C; Liver cancer due to hepatitis B; Liver cancer due to hepatitis C	Health center	*	
HC24	As resources permit, hepatitis B vaccination of high-risk populations, including healthcare workers, PWID, MSM, household contacts, and persons with multiple sex partners	Acute hepatitis B; Cirrhosis and other chronic liver diseases due to hepatitis B; Liver cancer due to hepatitis B	Health center	*	*
HC3	Management of preterm premature rupture of membranes, including administration of antibiotics	Maternal sepsis and other maternal infections; Indirect maternal deaths; Late maternal deaths; Neonatal preterm birth; Neonatal sepsis and other neonatal infections	Health center		
HC31	Focused use of vaccines for endemic infections, such as dengue, JEV, typhoid, meningococcus, and others	Typhoid and paratyphoid; Dengue; Yellow fever; Encephalitis	Health center	*	*
HC32	Provision of insecticide-treated nets to children and pregnant women attending health centers	Malaria, Dengue; Zika; Encephalitis; Yellow fever	Health center	*	
HC37	Low-dose inhaled corticosteroids and bronchodilators for asthma and for selected patients with COPD	Chronic obstructive pulmonary disease; Asthma	Health center	*	
HC49	Management of bipolar disorder using generic mood-stabilizing medications and psychosocial treatment	Bipolar disorder	Health center		*

Do LA, *et al. BMJ Global Health* 2021; 6:e005673. doi: 10.1136/bmjgh-2021-005673

HC54	Exercise programs for upper extremity injuries and disorders	Transport injuries; Unintentional injuries; Exposure to forces of nature; Other unintentional injuries; Exposure to mechanical forces; Foreign body; Self-harm and interpersonal violence; Other musculoskeletal disorders	Health center		*
HC62	Suturing laceration	Transport injuries; Unintentional injuries; Exposure to forces of nature; Other unintentional injuries; Exposure to mechanical forces; Foreign body; Self-harm and interpersonal violence	Health center		
HC63	Treatment of caries	Caries of deciduous teeth; Caries of permanent teeth	Health center		*
P1	Mass media messages concerning sexual and reproductive health and mental health for adolescents	HIV/AIDS and sexually transmitted infections; Depressive disorders; Bipolar Disorders; Anxiety disorders; Eating Disorders; Attention-deficit/hyperactivity disorder; Conduct disorder; Substance use disorders	Population-based		
P13	Mass media messages concerning awareness on handwashing and health effects of household air pollution	Diarrheal diseases; Acute hepatitis A; Acute hepatitis E; Chronic respiratory diseases	Population-based		
P3	Mass media messages concerning use of tobacco and alcohol	Alcohol abuse; Tobacco abuse	Population-based	*	
P4	Mass media encouraging use of condoms, voluntary medical male circumcision, and STI testing	HIV/AIDS and sexually transmitted infections	Population-based	*	

Do LA, *et al. BMJ Global Health* 2021; 6:e005673. doi: 10.1136/bmjgh-2021-005673

P5	Systematic identification of individuals with TB symptoms among high-risk groups and linkage to care ("active case finding")	Tuberculosis	Population-based	*	*
P6	Sustained vector management for Chagas disease, visceral leishmaniosis, dengue, and other nationally important causes of non-malarial fever	Chagas disease; Dengue; Visceral leishmaniasis; Typhoid and paratyphoid; Yellow fever; encephalitis	Population-based		
RH1	Full supportive care for preterm newborns	Neonatal disorders	Referral and specialty hospital		
RH10	Elective surgical repair of common orthopedic injuries (for example, meniscal and ligamentous tears) individuals with severe functional limitation	Transport injuries; Unintentional injuries; Exposure to forces of nature; Other unintentional injuries; Exposure to mechanical forces; Foreign body; Self-harm and interpersonal violence	Referral and specialty hospital		
RH11	Urgent, definitive surgical management of orthopedic injuries (for example, by open reduction and internal fixation	Transport injuries; Unintentional injuries; Exposure to forces of nature; Other unintentional injuries; Exposure to mechanical forces; Foreign body; Self-harm and interpersonal violence	Referral and specialty hospital		
RH13	Repair of club foot	Congenital musculoskeletal and limb anomalies	Referral and specialty hospital		
RH18	Surgery for trachomatous trichiasis	Trachoma	Referral and specialty hospital		

Do LA, *et al. BMJ Global Health* 2021; 6:e005673. doi: 10.1136/bmjgh-2021-005673

RH2	Specialized TB services, including management of MDR- and XDR-TB treatment failure and surgery for TB	Tuberculosis	Referral and specialty hospital	*	*
RH4	Management of acute ventilatory failure due to acute exacerbations of asthma and COPD; in COPD use of bi-level positive airway pressure preferred	Chronic obstructive pulmonary disease; Asthma	Referral and specialty hospital		
RH6	Use of percutaneous coronary intervention for acute myocardial infarction where resources permit	Ischemic heart disease	Referral and specialty hospital		
RH7	Treatment of early stage breast cancer with appropriate multimodal approaches (including generic chemotherapy), with curative intent, for cases that are detected by clinical examination at health centers and first-level hospitals	Breast cancer	Referral and specialty hospital		

Do LA, *et al. BMJ Global Health* 2021; 6:e005673. doi: 10.1136/bmjgh-2021-005673
